# Supplementary material for: Inferring internal states across mice and monkeys using facial features
Source: Nat Commun. 2025 Jun 4;16:5168. doi: 10.1038/s41467-025-60296-1 (PMC12137566; doi:10.1038/s41467-025-60296-1)
Supplement: Supplementary file 1 — Supplementary Information [file 41467_2025_60296_MOESM1_ESM.pdf]

Supplementary figures

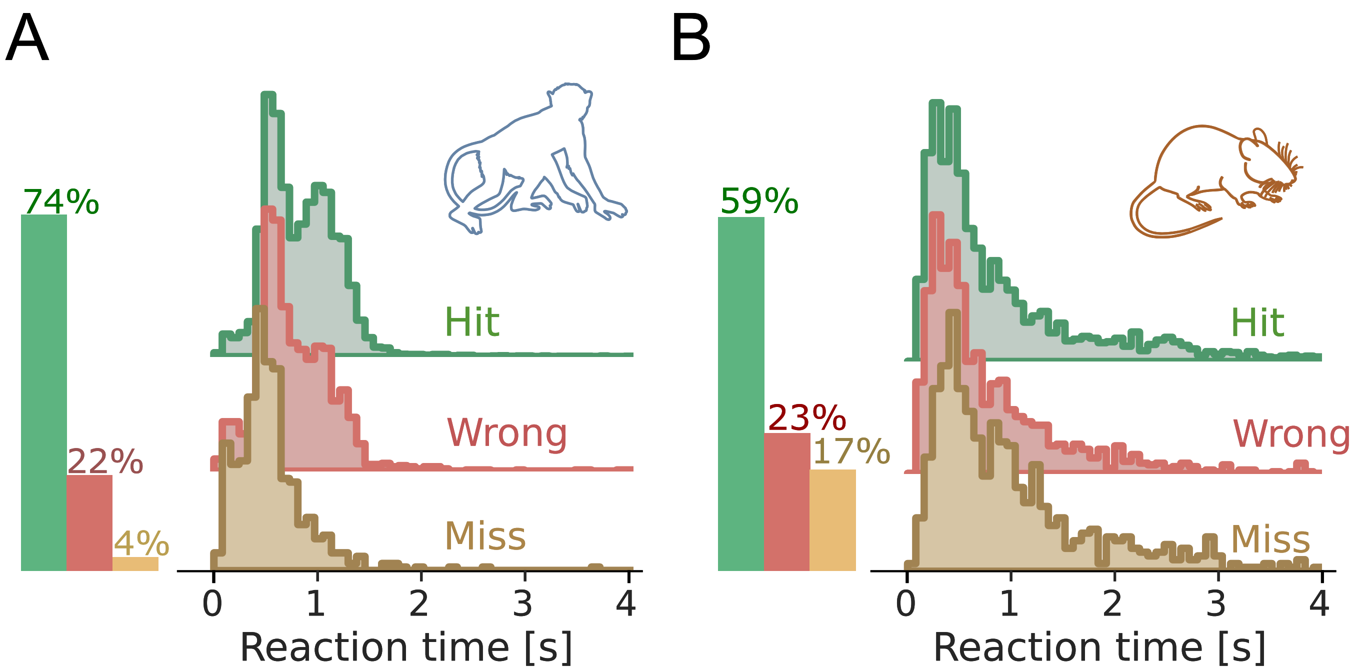

Figure S1. Reaction Times. Distribution of Reaction Times for macaques (A) and mice (B), split by behavioral outcome; data are pooled over sessions (n = 18 and n = 28 for macaques and mice, respectively). The three distributions largely overlap.

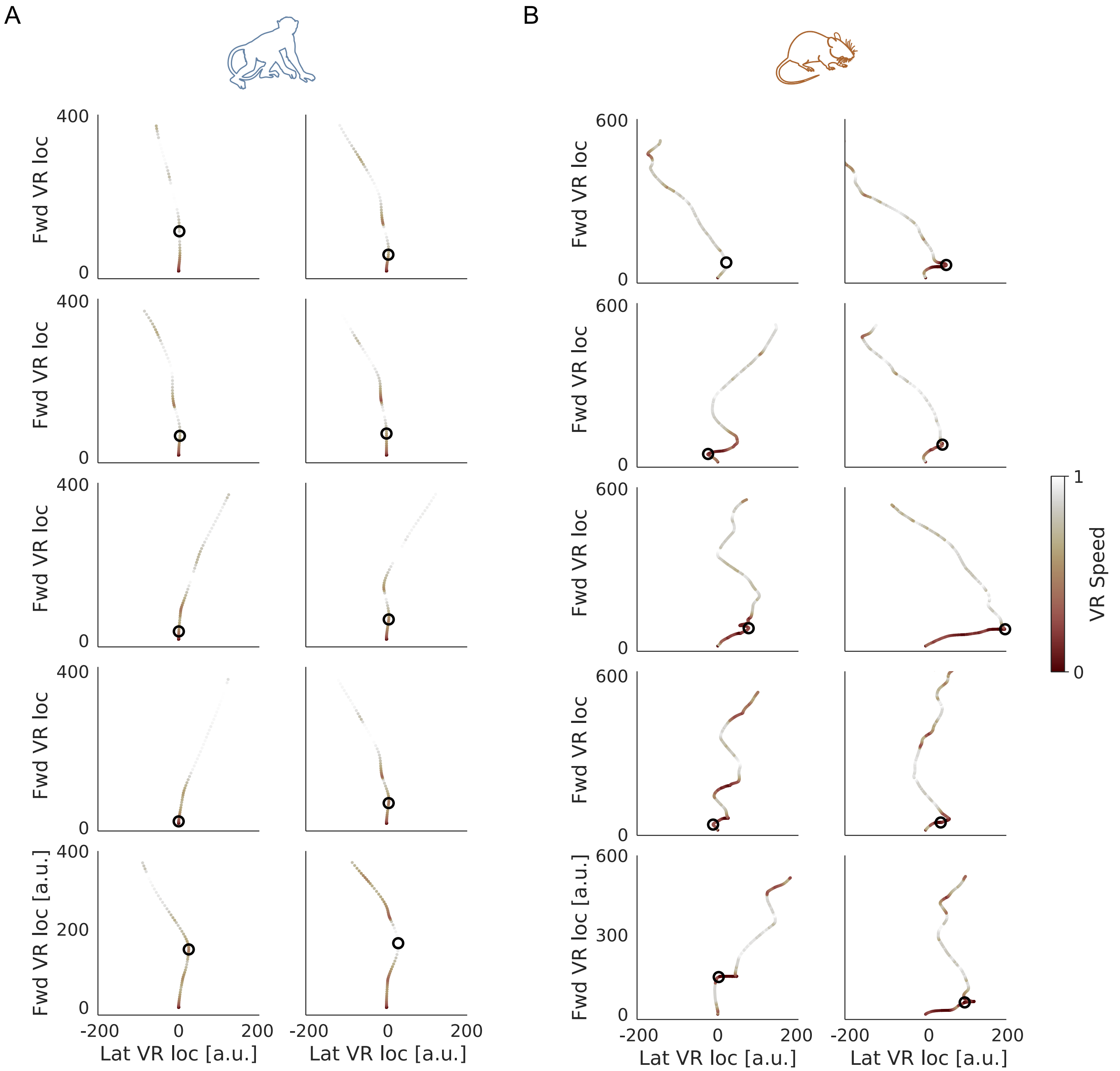

Figure S2. **Example VR paths** Paths are colored according to the normalized running speed. **A)** Example paths for macaques, with the detected RT as circles. **B)** Same, but for mice.

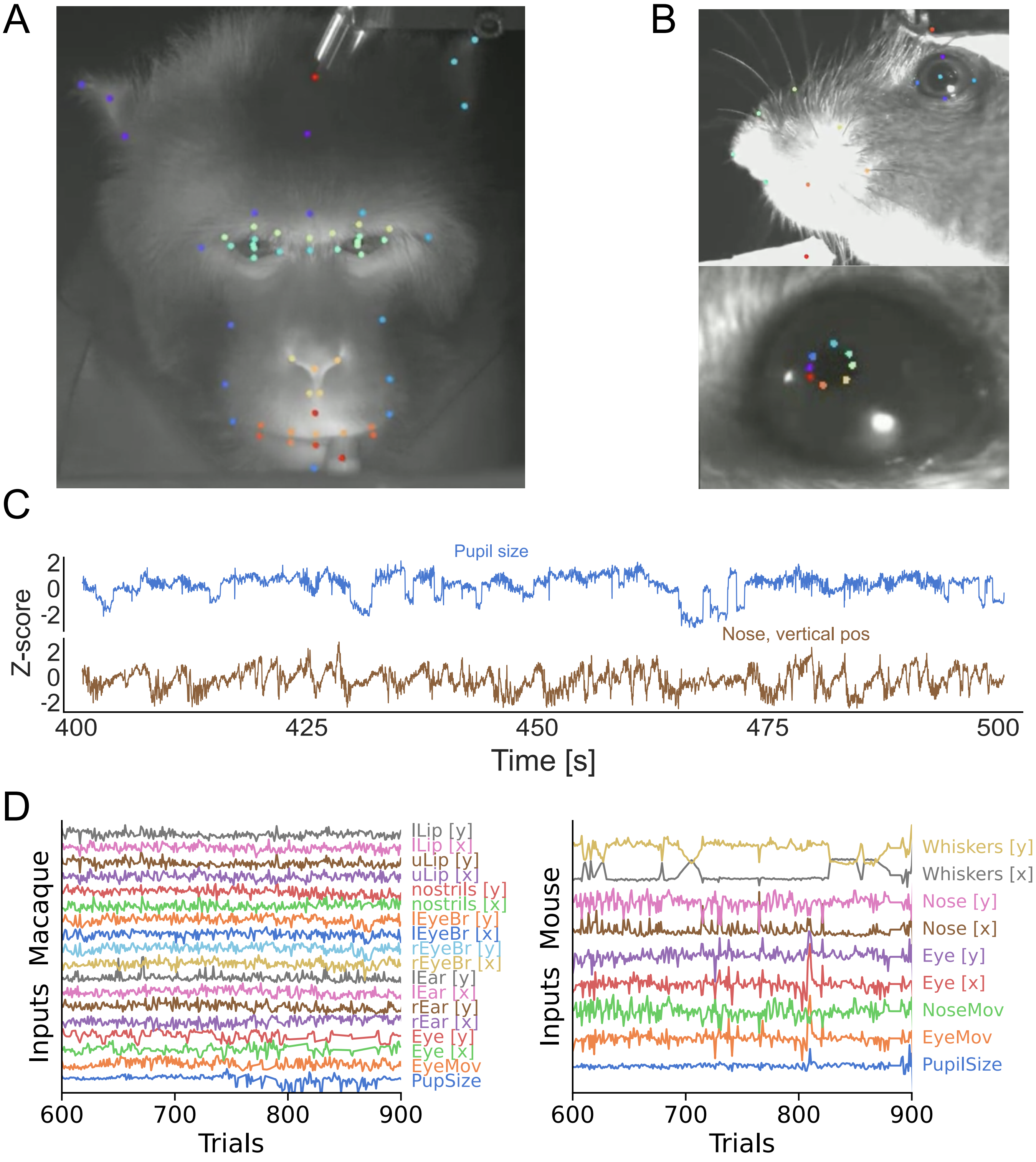

Figure S3. Face features. A) Example frame of the macaque face camera. We have marked the key points that we used as the raw data for our pipeline. For this animal, we track a total of 73 key points. Some of them will be aggregated into centroids of interest, to minimize the influence of noise. **B)** Same as **A)**, but for the mouse. In this case, we also have a separate model for tracking pupil changes. **C)** Two example traces of a common feature for both species, over time. **D)** As described in the Methods, we use trial summaries for each of the face features of interest. Here, we show all of them, after having preprocessed them, for an arbitrary selection of 300 trials.

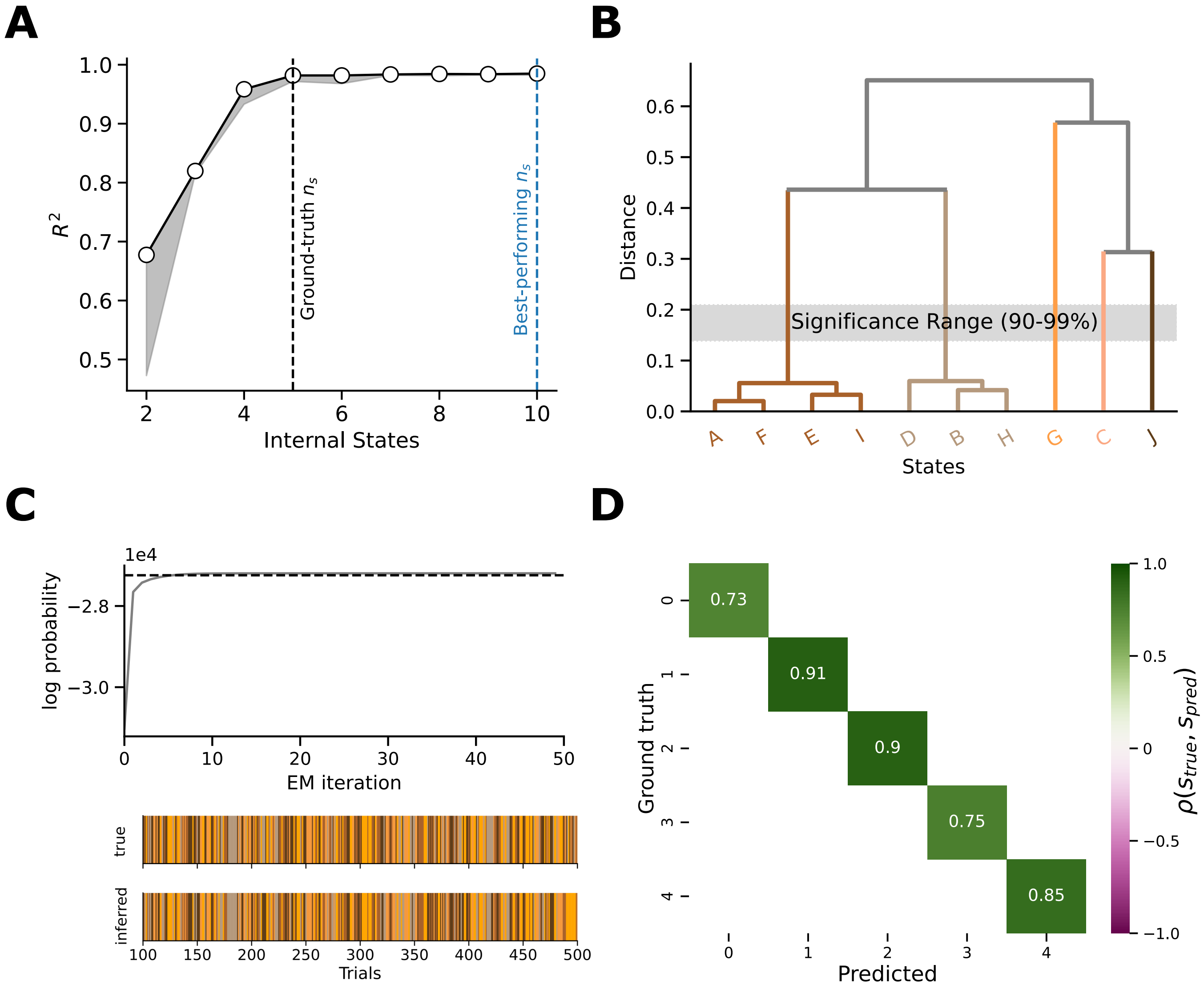

Figure S4. Synthetic states and emissions. A) Performance when varying the number of states. We are able to recover the number of states (vertical dashed line) that generated the ground truth emissions. **B)** For the selected number of states, log-probability of the fitted parameters; it approaches to the ground truth value (horizontal dashed line). **C)** Some example trials for the true and predicted states. State transitions are correctly captured. **D)** Temporal correlation between the one-hot encoded state arrays. There is a very close match between the predicted and the true states arrays.

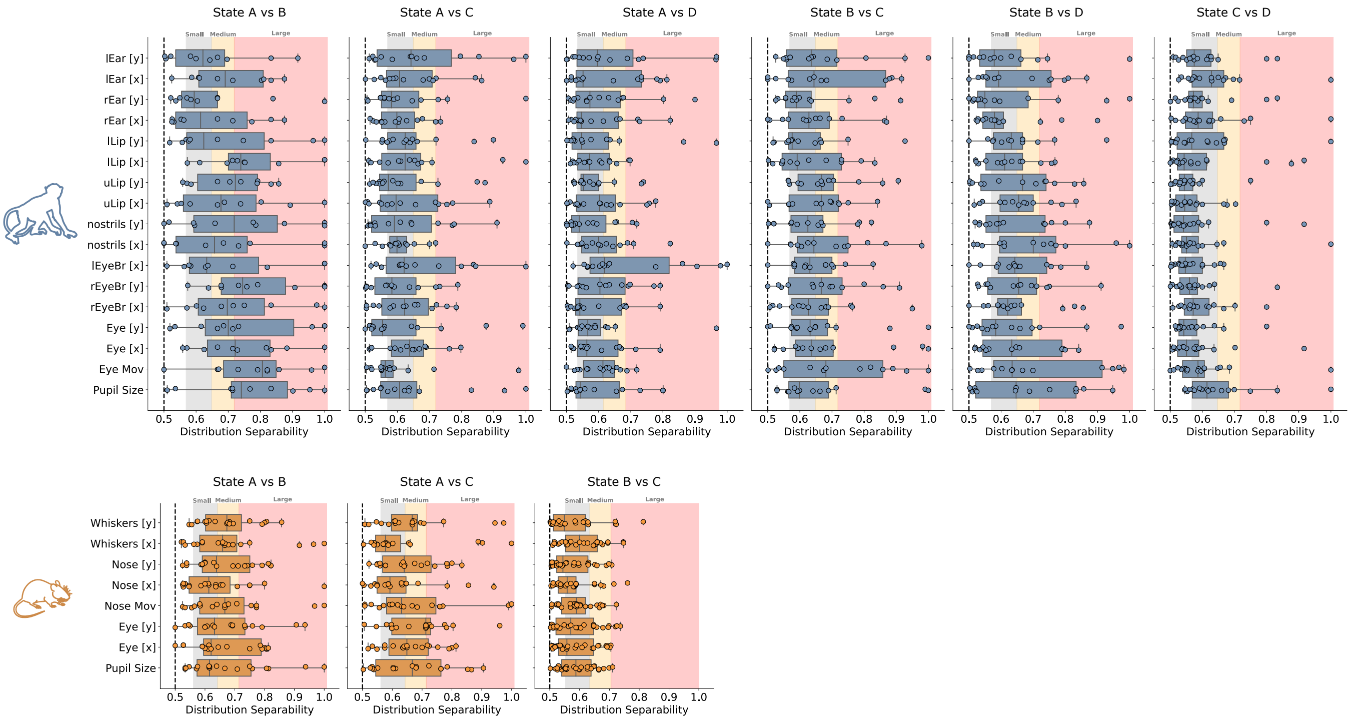

Figure S5. Face features separability across states. We quantify how dissimilar the different distributions of facial features are when split over states. Mathematically, this measure is defined as the Vargha-Delaney's A-statistic (also known as measure of stochastic superiority). This measure is bounded between 0.5 (total distribution overlap) and 1 (no overlap). We have shaded regions according to common interpretations [Vargha and Delaney(2000)]: Small separability (gray): $0.56 < \Omega < 0.64$; Medium separability (orange): $0.64 < \Omega < 0.71$; High separability (red): $\Omega \geq 0.71$. Each dot is an experimental session. No single feature appears useful on its own to disambiguate between states.

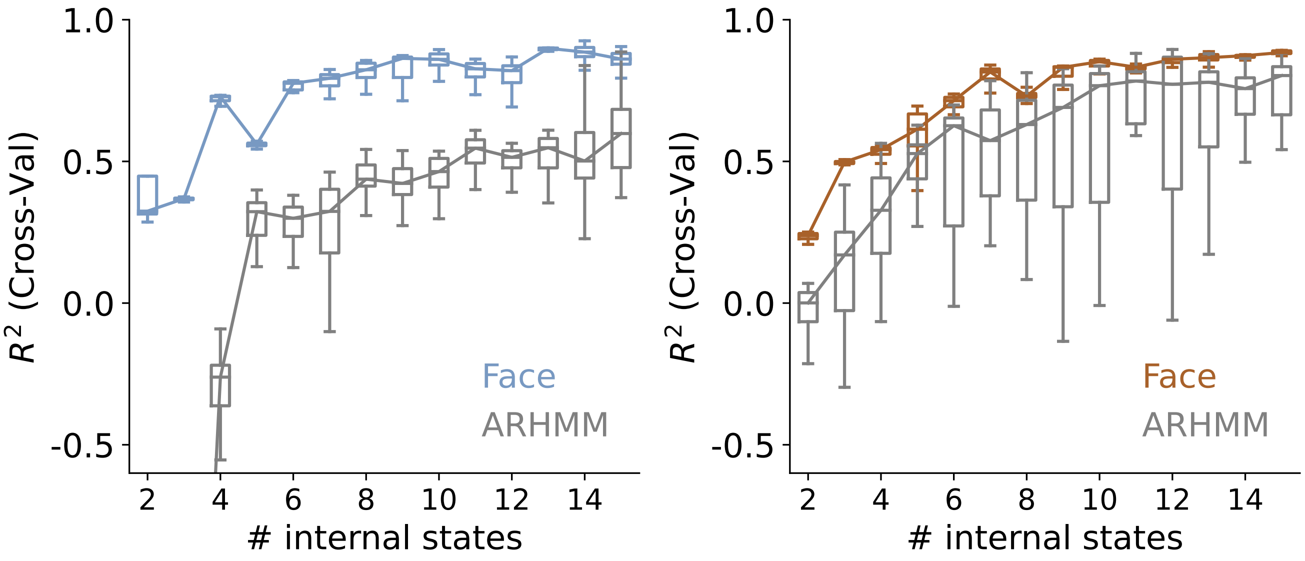

Figure S6. Comparison of the MSLR face model and the reaction time Auto-Regressive HMM. Both face feature models outperform their Autoregressive counterparts, for any number of internal states that we swept over. Nevertheless, it can be seen that the performance gap is smaller in mice than in macaques. This is consistent with the finding that mice are more history dependent than macaques (See Fig. 5 D).

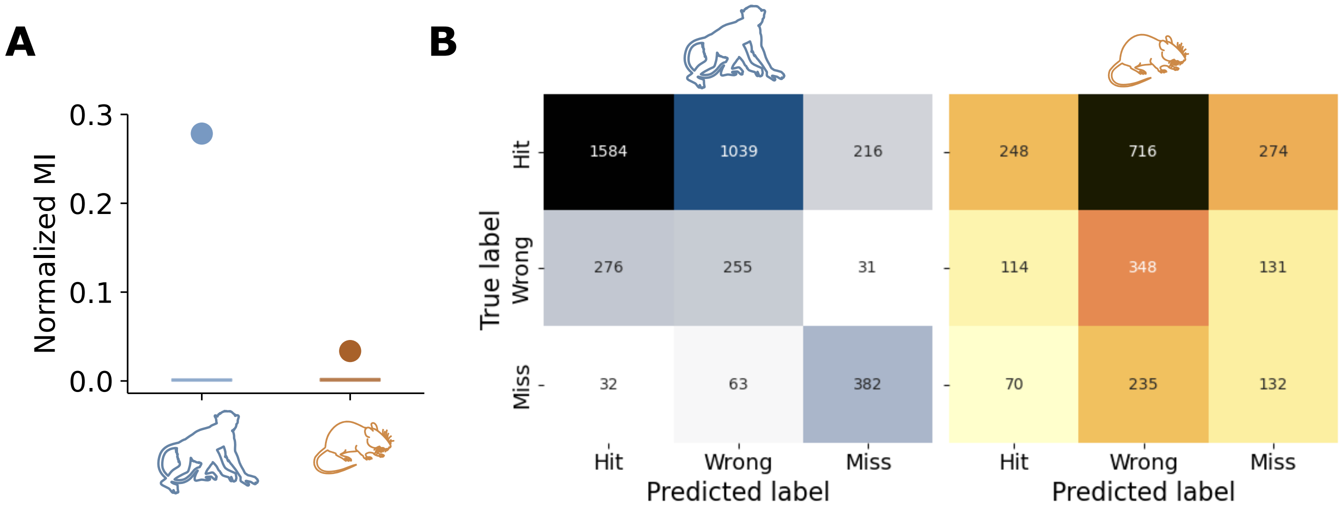

Figure S7. Inferred state probabilities decode outcome beyond chance. We used a normalized version of Mutual Information (panel A) that already takes chance level into account and sets that as 0. The filled dots are the actual values and the shaded regions represent the surrogate distributions (built by randomly shuffling the outcomes over trials). As expected by construction of the metric, the random prediction is precisely at 0, whereas the measured value for both species is non-zero. When identifying which states were confused for another state by the model (panel B), we see that wrong and hit trials are misclassified more regularly than both of these trial types with miss trials. As before, for mice, wrong trials appear to be confused roughly equally for hit and miss trials, while in monkeys they are almost exclusively confused for hit trials, again suggesting that in monkeys hit and wrong states are more similar to each other than in mice.

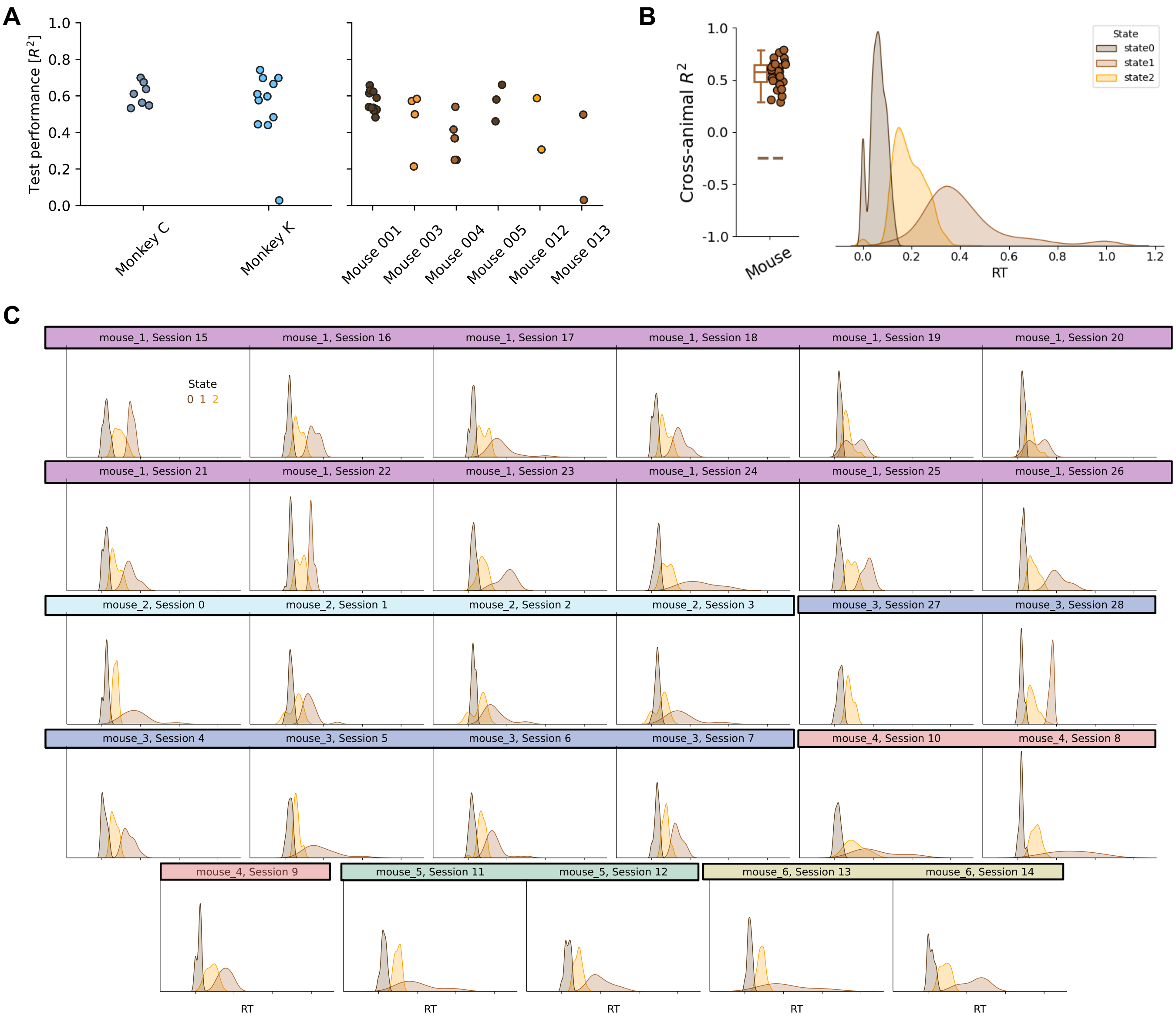

Figure S8. Model generalization across animals. A) We show that the model performs uniformly well across animals, for both species (left, macaques; right, mice). There is no outlier animal for which the model does not perform well or just a group of them for which it does. **B)** If we follow a Leave-One-Out Cross-Validation (i.e., training a model with $n-1$ mice and testing on another one), we see that the model generalizes and there is a robust mapping of states to Reaction Times. **C)** We also show individual sessions, grouped by *validation mouse*.

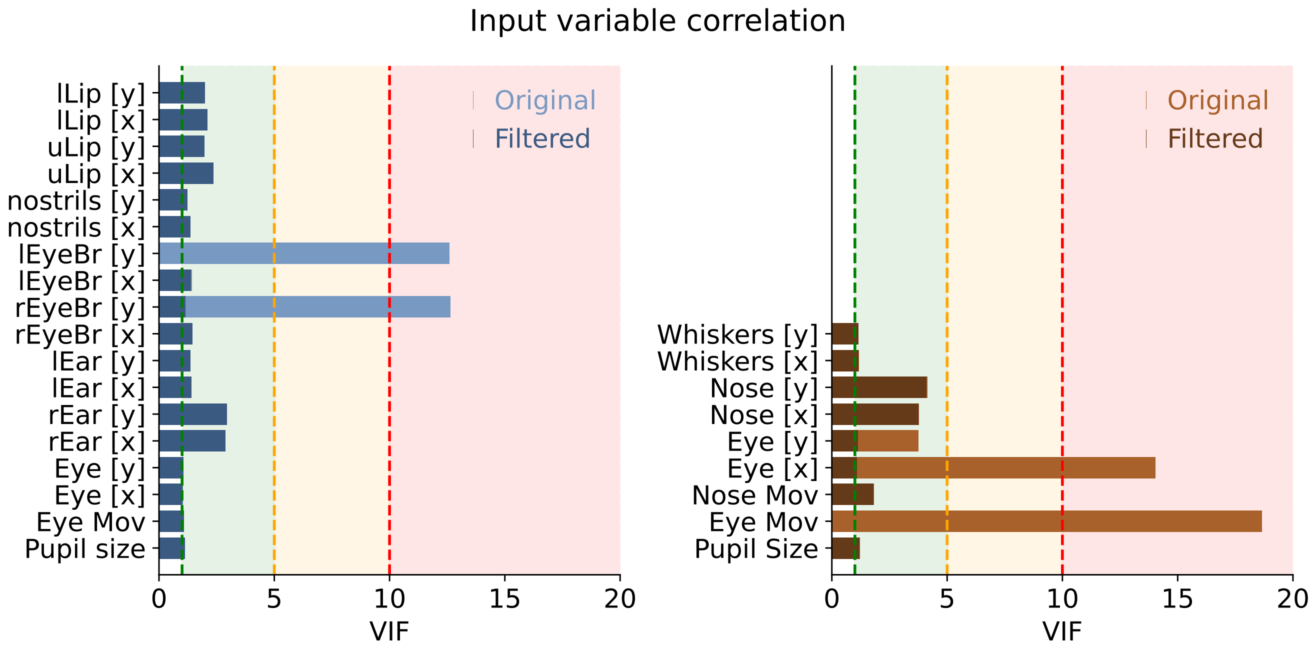

Figure S9. Input variable correlation. We show that, out of all of the original variables (in lighter colors), we end up discarding one per animal (*Left Eyebrow [y], macaques; Eye movement, mice*), given that they were highly multi-colinear with some of the other predictors, as measured by the Variance Inflation Factor (VIF). After discarding them and recomputing the VIF, we did not find any alarming colinearity.

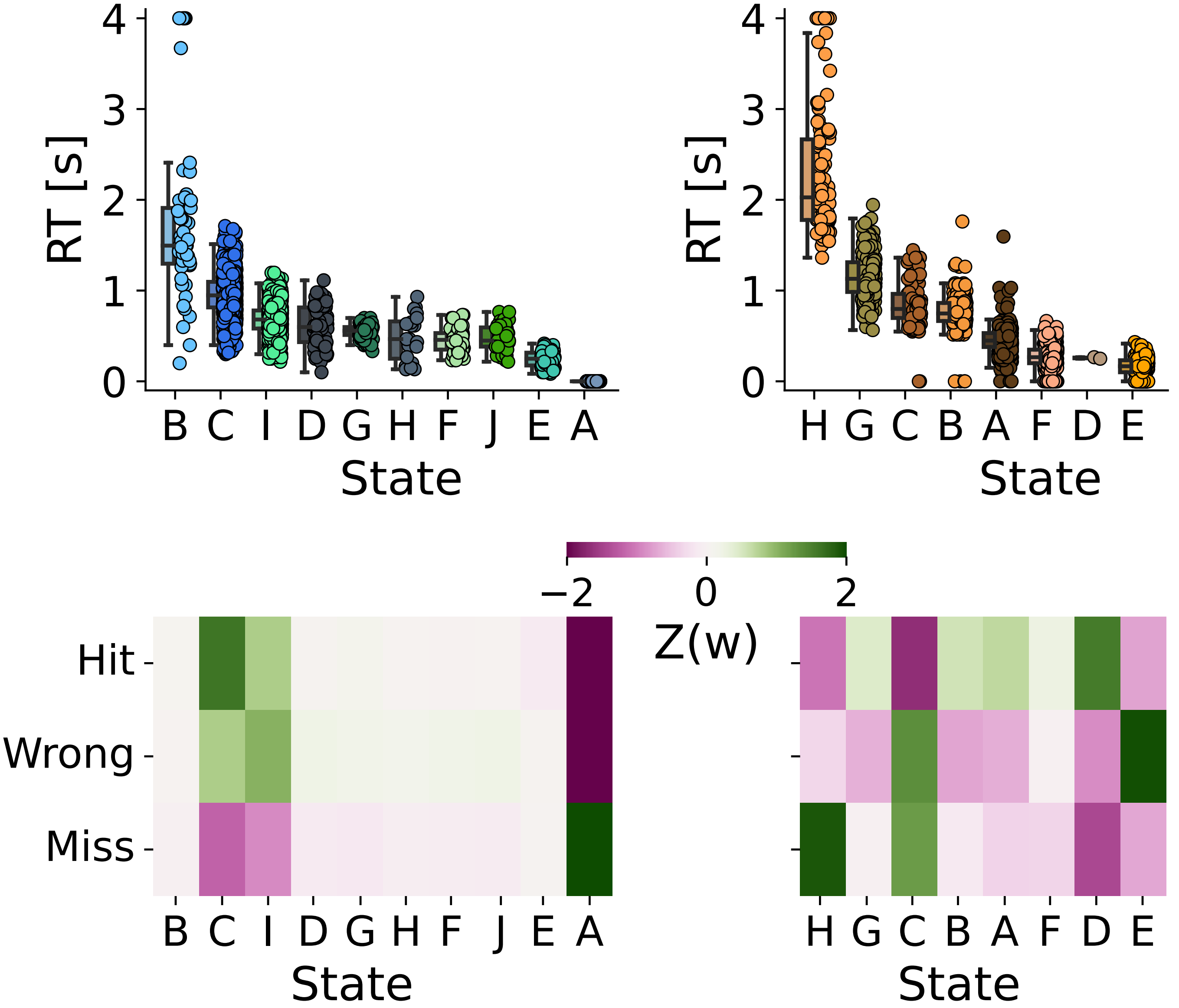

Figure S10. Fragmented states. We show that, if we select the number of states (n_s) based on CV performance only (Fig. 2 in the main text), we would use $n_s = 10$ and $n_s = 8$ for macaque and mice, respectively. When looking at the emissions and the correlation with outcomes for each of these states, we find what others have already reported in the context of HMMs [Fox et al.(2007)Fox, Sudderth, Jordan, and Willsky]: if we allow states to be too fragmented, they will become redundant. In our case, we see that emissions and correlation with outcomes are highly redundant across states. Hence, both because of these reasons because of the state clustering results (Fig. S11 A), we decided to reduce the number of states for the rest of the paper.

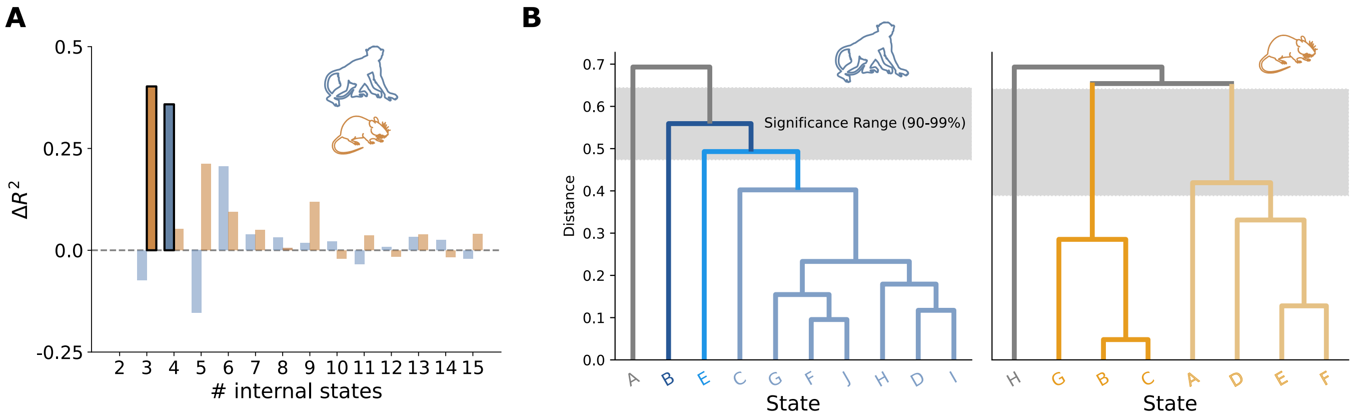

Figure S11. State selection pipeline. We selected the number of states following the same pipeline as in Fig. S4: we first select the n_s corresponding to the highest CV R^2 ($n_s = 10, 8$ for macaques and mice, respectively); then, we inspect whether the corresponding emissions (Fig. S10) can be clustered together. This yields a potential $n_s \in \{2, 3, 4\}$ for macaques and $n_s \in \{2, 3, 4\}$ for mice (panel A). In order to disambiguate between these, we compute the finite difference in CV R^2 (panel B). We end up selecting $n_s = 4, 3$ for macaques and mice, respectively

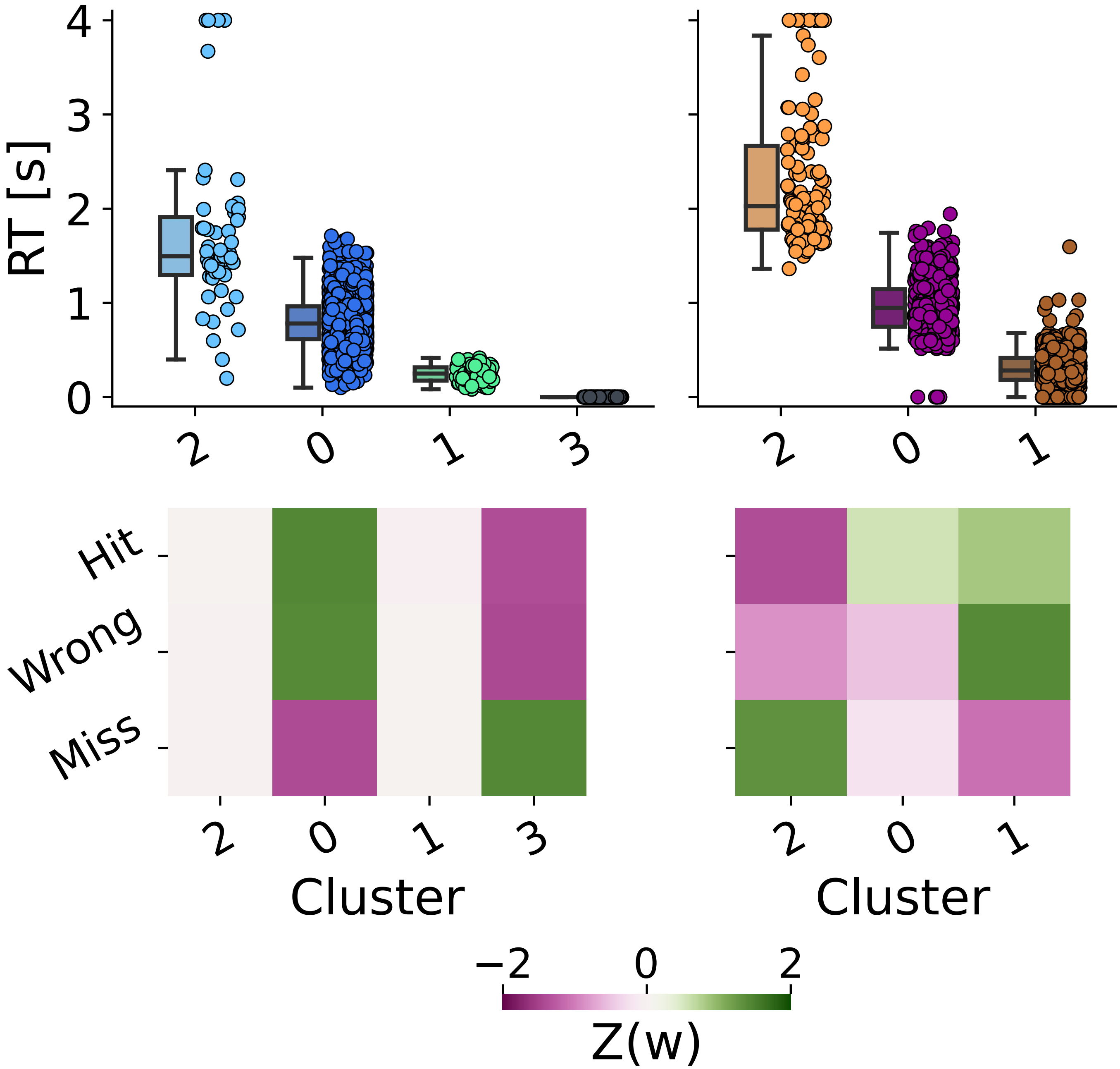

Figure S12. Clustered states. If we cluster the emission distributions corresponding to the fragmented states we found in Fig. S10, we see that the most salient features demonstrated in the main figures are reproduced here: States cluster onto specific ranges of reaction times from slow to fast, and they spontaneously map onto trial outcomes, with hit and wrong trials being more interchangeable than miss trials with either hit or wrong trials. This is remarkable because these states and the ones from the main text do not need to be inferred from the same local minima in the loss landscape.

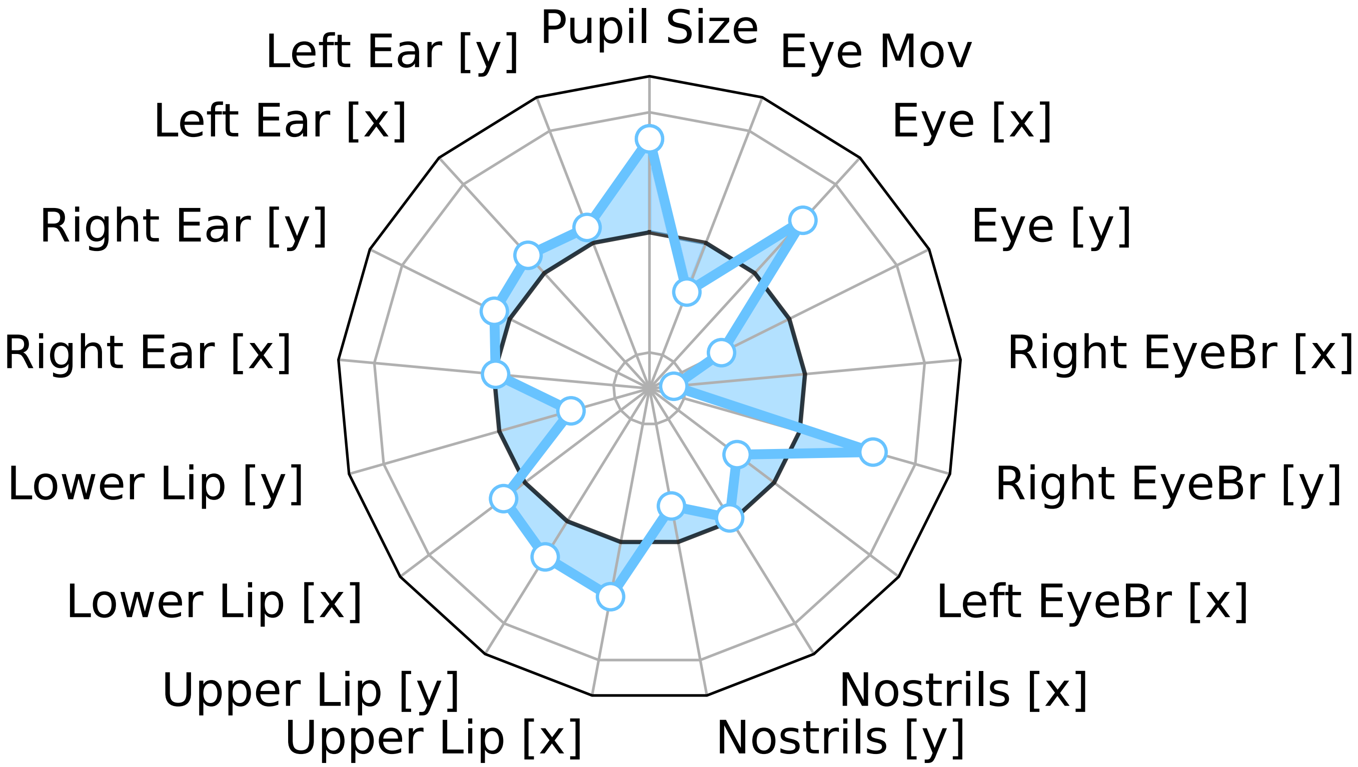

Figure S13. Face features importance for State B in macaques. In comparison to the other three internal states in macaques, this state is associated with yet a different constellation of facial features.

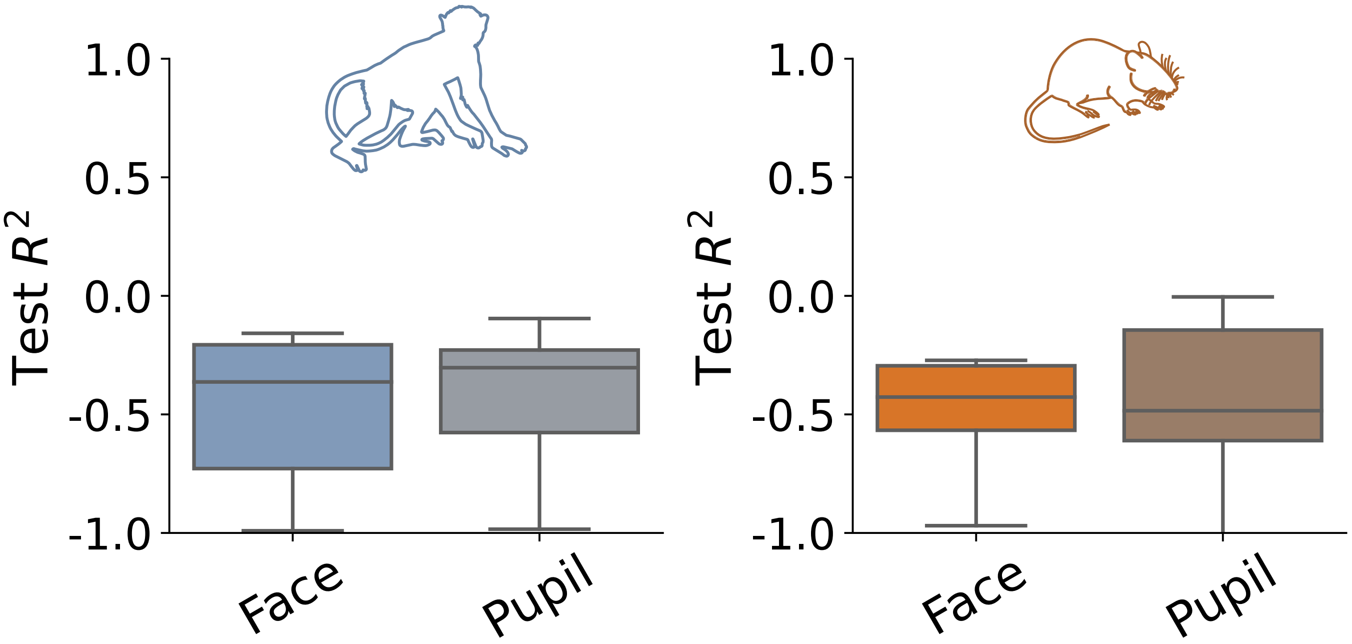

Figure S14. No-switching model performances. If we assume there is a simple linear relationship (i.e. we do not allow for any state switching) between the face (or the pupil) and the Reaction Time, the test performance remains at chance level for monkeys (left) and mice (right).

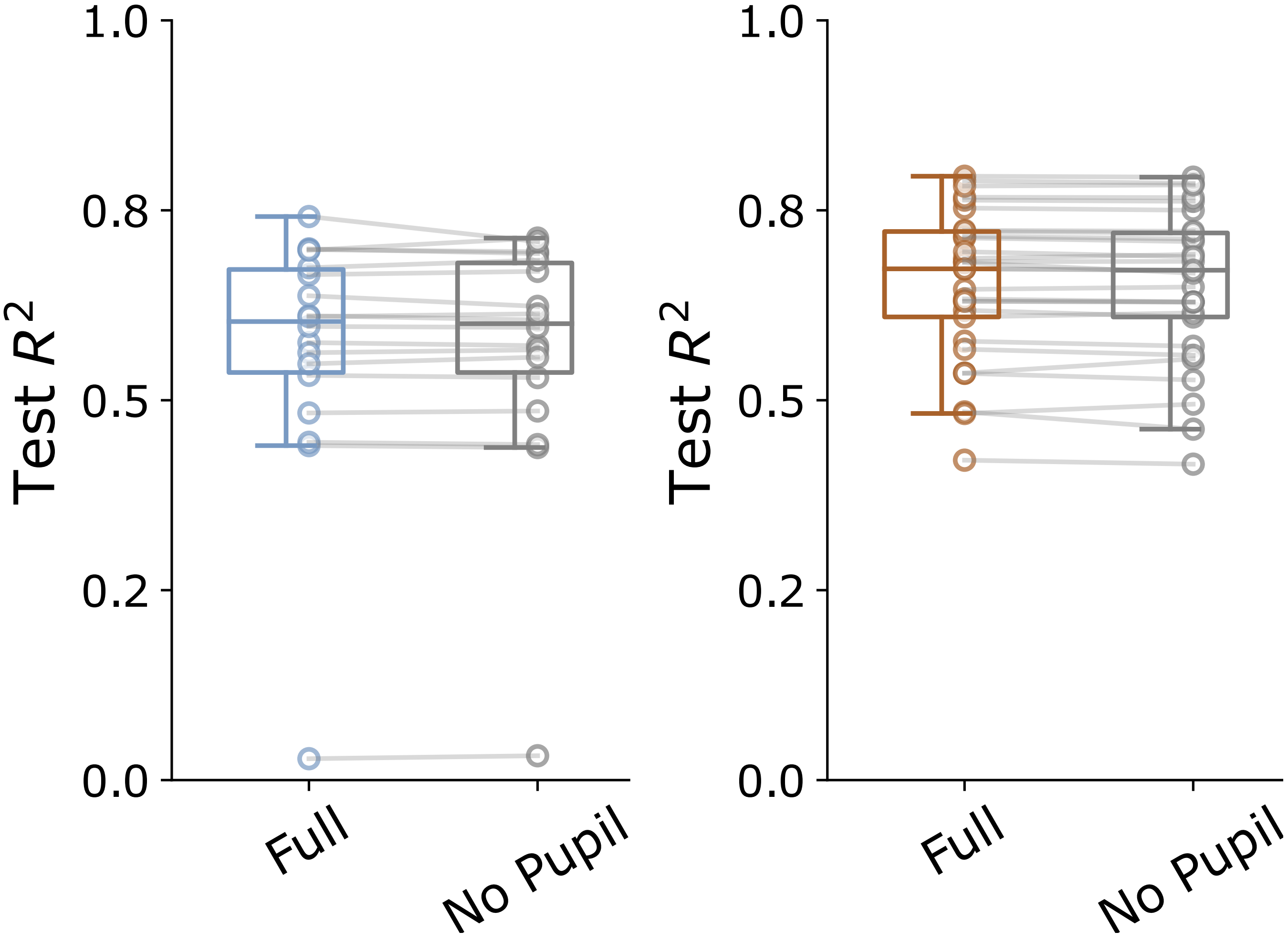

Figure S15. Unaltered model performance without pupil size. If we remove the pupil size as a predictor for Reaction Time, model performance remains unchanged. This speaks against the usual perspective by which pupil size is paramount in inferring internal states.

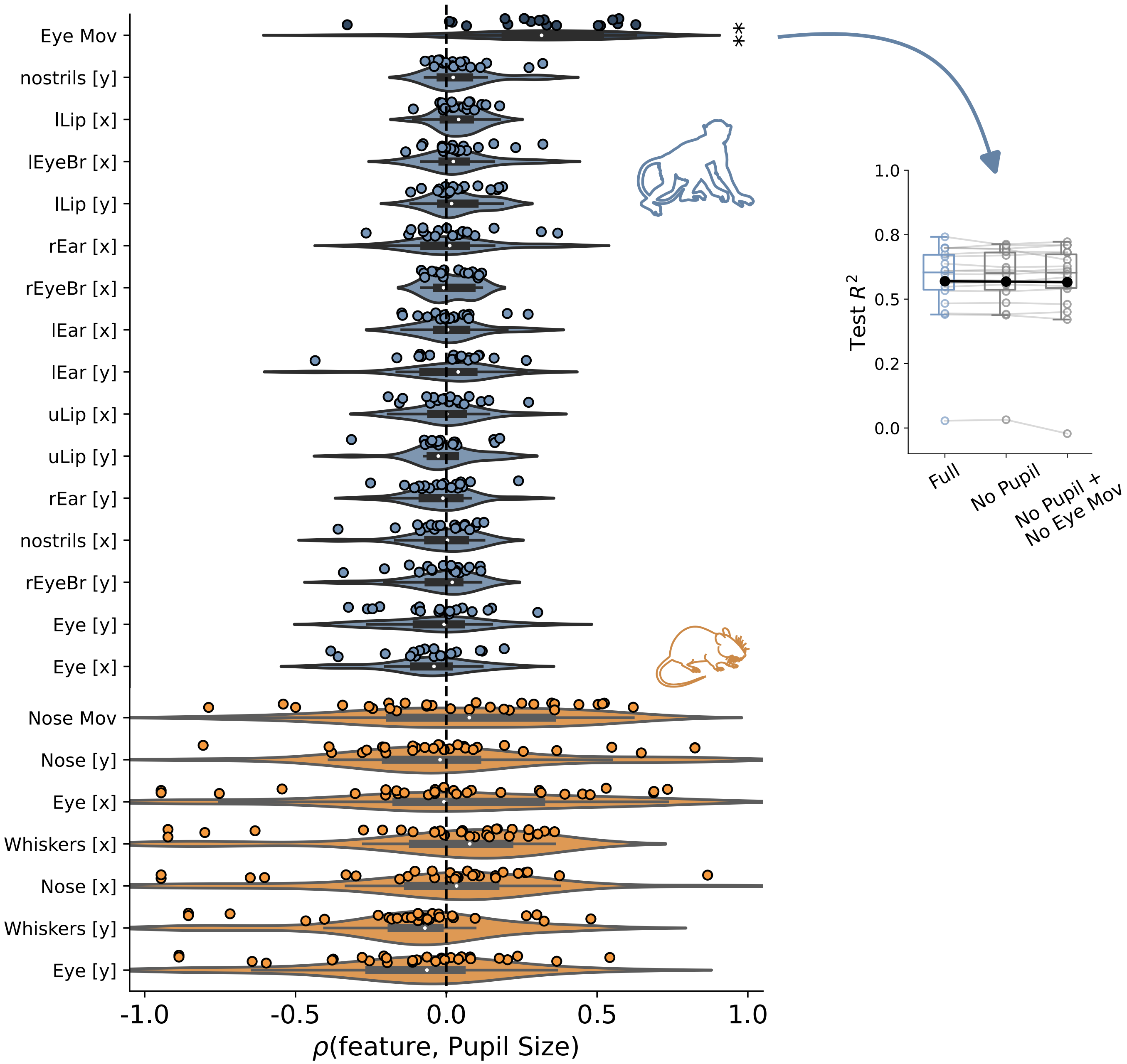

Figure S16. Unaltered model performance without pupil size and correlated features. If, on top of removing the pupil size variable, we also remove all predictors that are significantly correlated with it, the model performance remains unchanged. Left panel: Distribution of joint probabilities between pupil size and all other facial features. Only eye movement is significantly related to pupil size. Panel inset on the right: Model performance for the full model, model with pupil size removed, and with pupil size and eye movement removed. Model performance remains noticeably stable. This, together with Fig. S15 speaks against the usual perspective by which pupil size is paramount in inferring internal states.

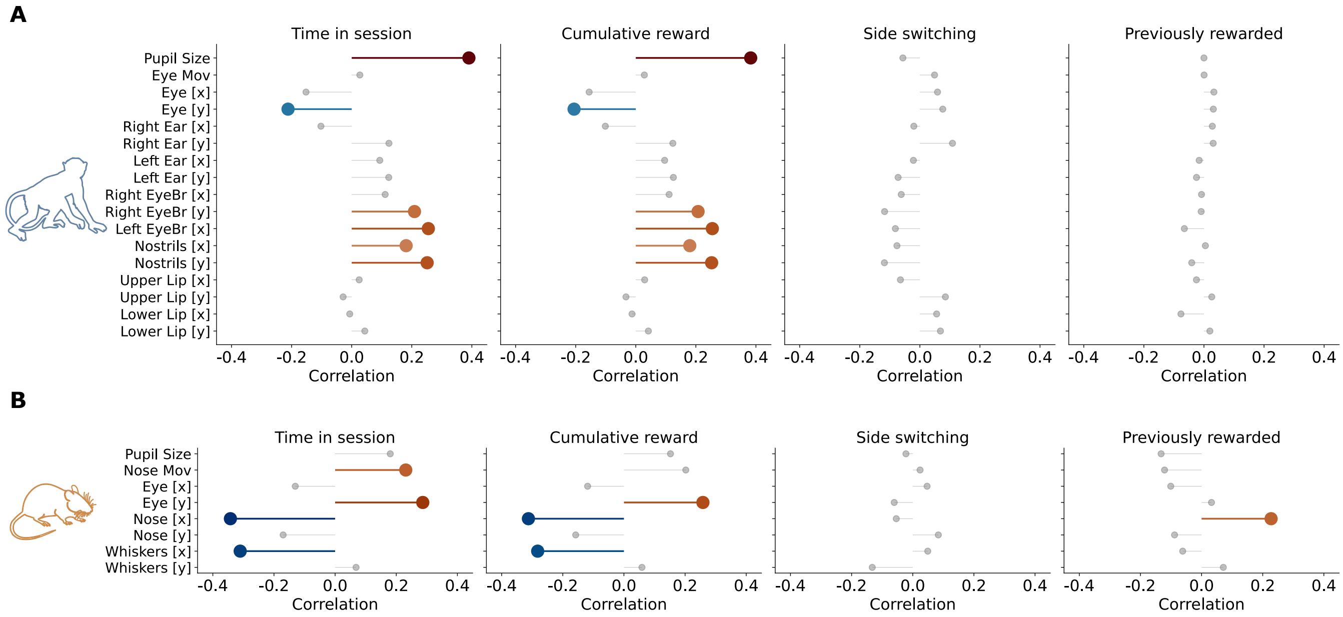

Figure S17. Facial features and task history correlations, for macaques (A) and mice (B): time spent in session (left), cumulative reward in the session (center left), the correct stimulus switching location compared to the previous trial (center right), and whether the previous trial had been rewarded or not (right). Weights highlighted in colour are significantly correlated to the subsequent trial outcome.

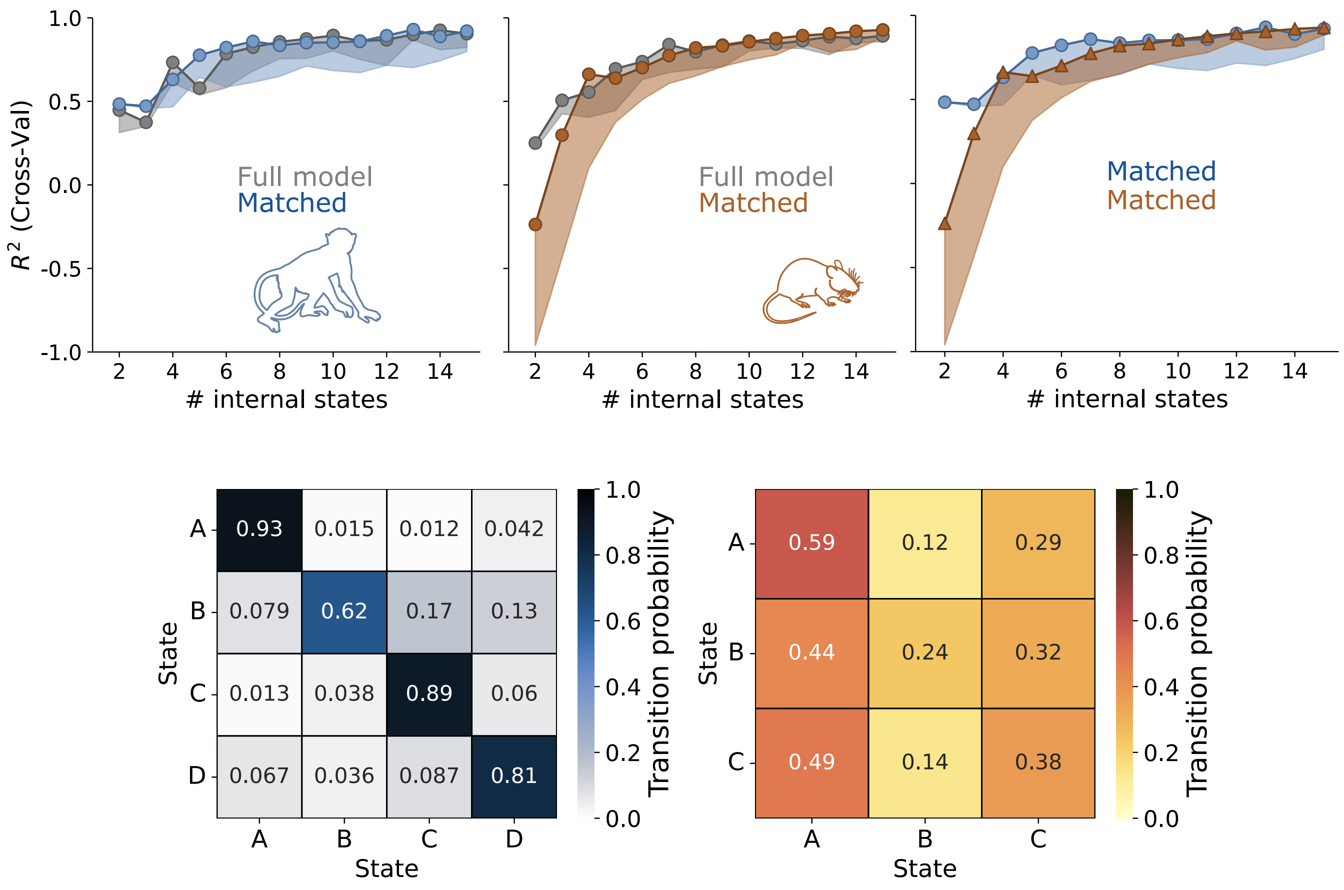

Figure S18. Matched models. Top panels: Model performance across different numbers of states when models are trained on a data set in which we match the number of predictors, trials and animals we use across both species. Bottom panels: Transition matrices between states for both species when models are trained on matched data. Transition matrices show the same qualitative dynamics as the models trained on non-matched data sets.

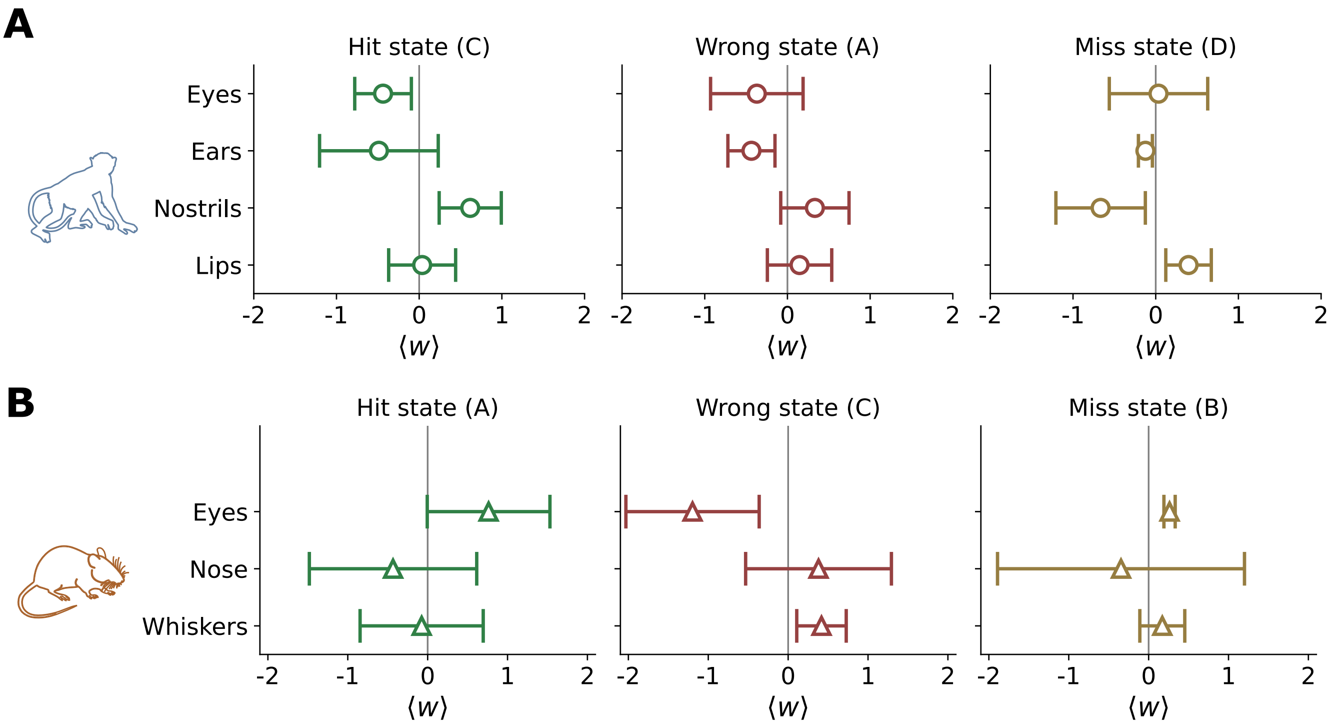

Figure S19. Simplified face features weights. Collapsing facial features into a few general groups such as eyes, ears, and nose, again shows varying contributions of these feature groups for the different states. We show the signed $L1$ norm of the grouped feature weights for monkeys (A) and mice (B) across hit, miss and wrong states.

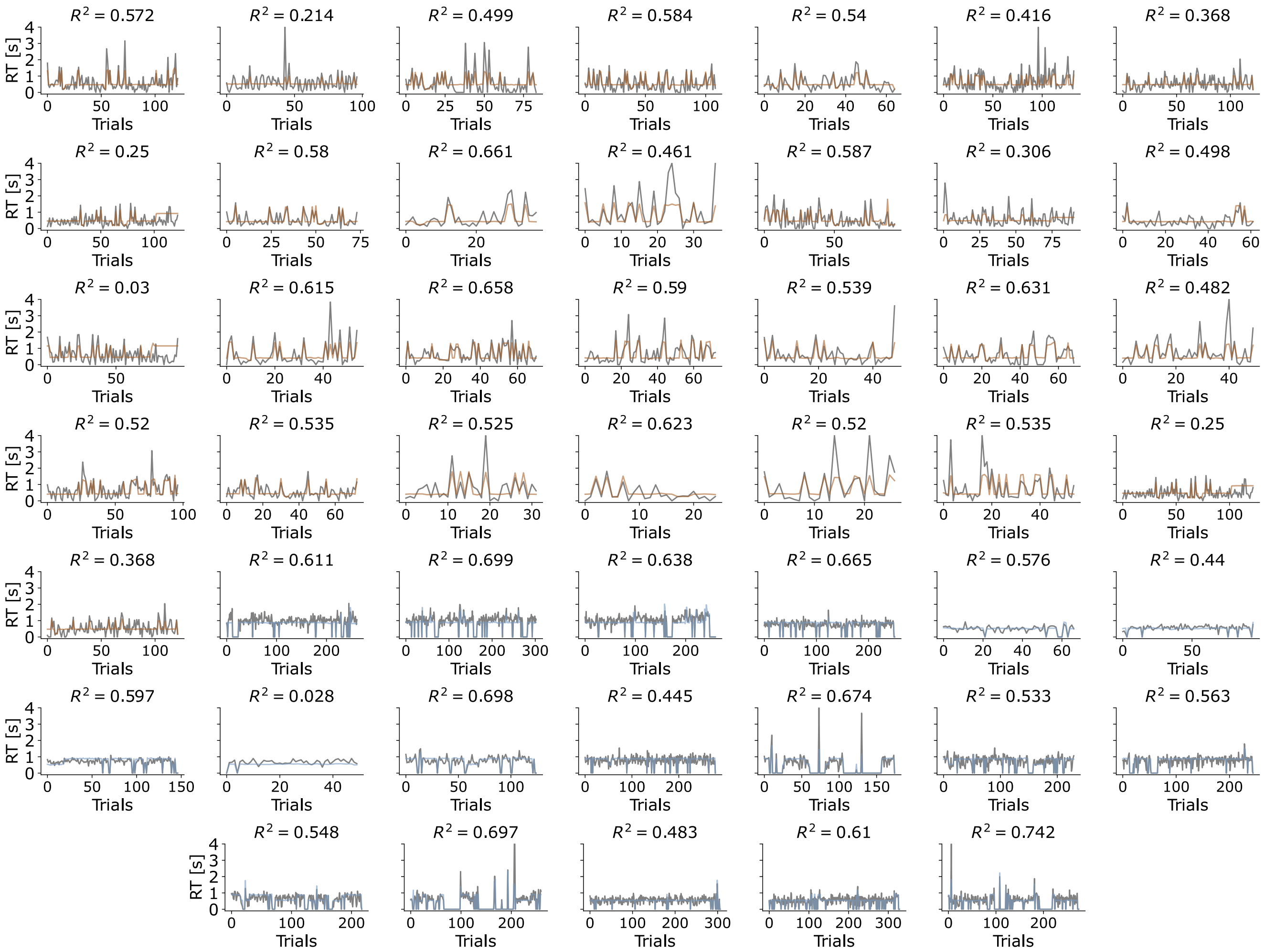

Figure S20. Predicted and true RTs for all sessions, for the held out test set. For both species, true RTs are shown in gray; for mice, predicted RTs are shown in orange; for macaques, predicted RTs are shown in blue. Each subplot is titled with that session's model performance (R^2).

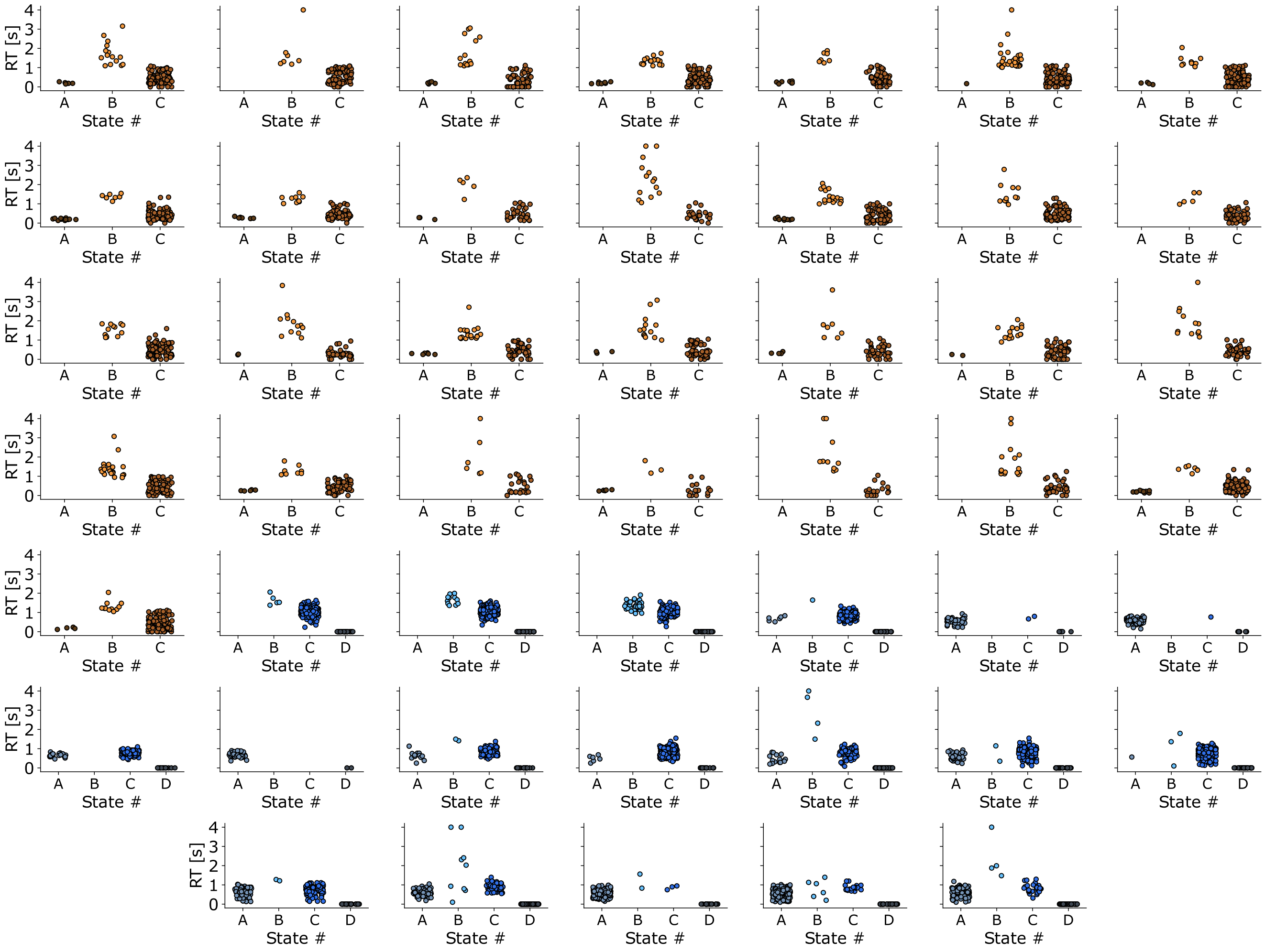

Figure S21. RTs over states for all sessions, for the held out test set. For mice, distributions are shown in orange; for macaques, distributions are shown in green-blue.

Supplementary References

References

[Vargha and Delaney(2000)] András Vargha and Harold D Delaney. A critique and improvement of the cl common language effect size statistics of mcgraw and wong. *Journal of Educational and Behavioral Statistics*, 25(2): 101–132, 2000.

[Fox et al.(2007)Fox, Sudderth, Jordan, and Willsky] Emily B Fox, Erik B Sudderth, Michael I Jordan, and Alan S Willsky. The sticky hdp-hmm: Bayesian nonparametric hidden markov models with persistent states. *Arxiv preprint*, 2, 2007.
